# Totipotency of mouse zygotes extends to single blastomeres of embryos at the four-cell stage

**DOI:** 10.1038/s41598-021-90653-1

**Published:** 2021-05-27

**Authors:** Marino Maemura, Hiroaki Taketsuru, Yuki Nakajima, Ruiqi Shao, Ayaka Kakihara, Jumpei Nogami, Yasuyuki Ohkawa, Yu-ichi Tsukada

**Affiliations:** 1 Graduate School of Systems Life Sciences, Kyushu University, 744 Motooka, Nishi-ku, Fukuoka, 819-0395 Japan; 2 Advanced Biological Information Research Division, INAMORI Frontier Research Center, Kyushu University, 744 Motooka, Nishi-ku, Fukuoka, 819-0395 Japan; 3 Division of Transcriptomics, Medical Institute of Bioregulation, Kyushu University, 3-1-1 Maidashi, Higashi-ku, Fukuoka, 812-8582 Japan

**Keywords:** Developmental biology, Molecular biology

## Abstract

In multicellular organisms, oocytes and sperm undergo fusion during fertilization and the resulting zygote gives rise to a new individual. The ability of zygotes to produce a fully formed individual from a single cell when placed in a supportive environment is known as totipotency. Given that totipotent cells are the source of all multicellular organisms, a better understanding of totipotency may have a wide-ranging impact on biology. The precise delineation of totipotent cells in mammals has remained elusive, however, although zygotes and single blastomeres of embryos at the two-cell stage have been thought to be the only totipotent cells in mice. We now show that a single blastomere of two- or four-cell mouse embryos can give rise to a fertile adult when placed in a uterus, even though blastomere isolation disturbs the transcriptome of derived embryos. Single blastomeres isolated from embryos at the eight-cell or morula stages and cultured in vitro manifested pronounced defects in the formation of epiblast and primitive endoderm by the inner cell mass and in the development of blastocysts, respectively. Our results thus indicate that totipotency of mouse zygotes extends to single blastomeres of embryos at the four-cell stage.

## Introduction

Reproduction is a fundamental feature of all known organisms. In mammals, heterogametes consisting of oocytes and sperm undergo fusion during fertilization, and the resulting zygote initiates a developmental program that gives rise to a new individual comprised of a diverse range of cell types. The ability of zygotes to produce a fertile adult from a single cell when placed in a supportive environment (for mammals, a uterus) is referred to as totipotency^1^. Preimplantation mammalian embryos at the stage prior to segregation of the first two cell lineages—the embryonic cell lineage (the inner cell mass, or ICM) and the extraembryonic cell lineage (the trophectoderm, or TE)—contain two distinct types of cell: totipotent cells and plenipotent cells, the latter of which are able to generate all derivative cells of both the ICM and TE but are unable to produce a fully formed individual from a single cell^1^. In contrast to totipotent cells, which are capable of generating a globally coordinated developmental sequence as a one-cell embryo, plenipotent cells are unable to organize their progeny cells into an integrated body plan. Plenipotent cells have been described as possessing totipotency in a less strict sense of the word^1–3^, but we use the term plenipotency in our study to avoid confusion caused by the two senses of the term totipotency as proposed^1^. After segregation of the ICM and TE in preimplantation embryos, cells in the ICM commit to two different lineages, epiblast (EPI) and primitive endoderm (PrE). EPI gives rise to most cells of the embryo proper and is the origin of pluripotent embryonic stem cells (ESCs), whereas PrE eventually differentiates into the parietal endoderm and visceral endoderm of the yolk sac. The ability of EPI cells to generate most cell types of the embryonic lineage but not those of the extraembryonic lineage is defined as pluripotency, which differs from both totipotency and plenipotency. The potency of cells in early embryos thus becomes restricted from totipotency to plenipotency to pluripotency as development proceeds.

Given that totipotent cells are the source of all multicellular organisms, a better understanding of totipotency may have a wide-ranging impact on biology. However, our knowledge of totipotency is limited relative to that of pluripotency. Substantial progress in the understanding of pluripotency has been due mostly to the study of ESCs. The molecular basis and key determinants of pluripotency, including transcriptional and epigenetic networks, have been extensively characterized in ESCs^4–6^. Knowledge obtained from these studies led to the development of methods for the generation of another type of pluripotent cell, the induced pluripotent stem cell (iPSC). The artificial introduction of important determinants of pluripotency thus induces the formation of iPSCs from terminally differentiated somatic cells^7,8^. Furthermore, insight into plenipotency has been advanced recently by the discovery of 2C-like ESCs^9^, extended pluripotent stem cells^10^, and expanded potential stem cells^11^. A fuller understanding of totipotency will therefore require the study of totipotent cells. However, the precise delineation of totipotent cells in mammals has remained unclear, with evidence suggesting that totipotent cells exist up to the two-cell stage (mouse^12^, rat^13^, and horse^14^), four-cell stage (cattle^15^ and monkey^16^), or eight-cell stage (rabbit^17^, sheep^18^, and pig^19^) of preimplantation embryos.

We have now evaluated the developmental potential of single blastomeres of preimplantation mouse embryos. We found that totipotent cells are present up to the four-cell stage of mouse embryos, and that pre- and peri-implantation developmental potential are essentially lost in single blastomeres of embryos at the morula or eight-cell stage, respectively. Our findings thus refine current knowledge of the totipotency and developmental potential of single blastomeres.

## Results

### Preimplantation developmental potential is essentially absent in single blastomeres isolated at the morula stage

**Figure 1:**
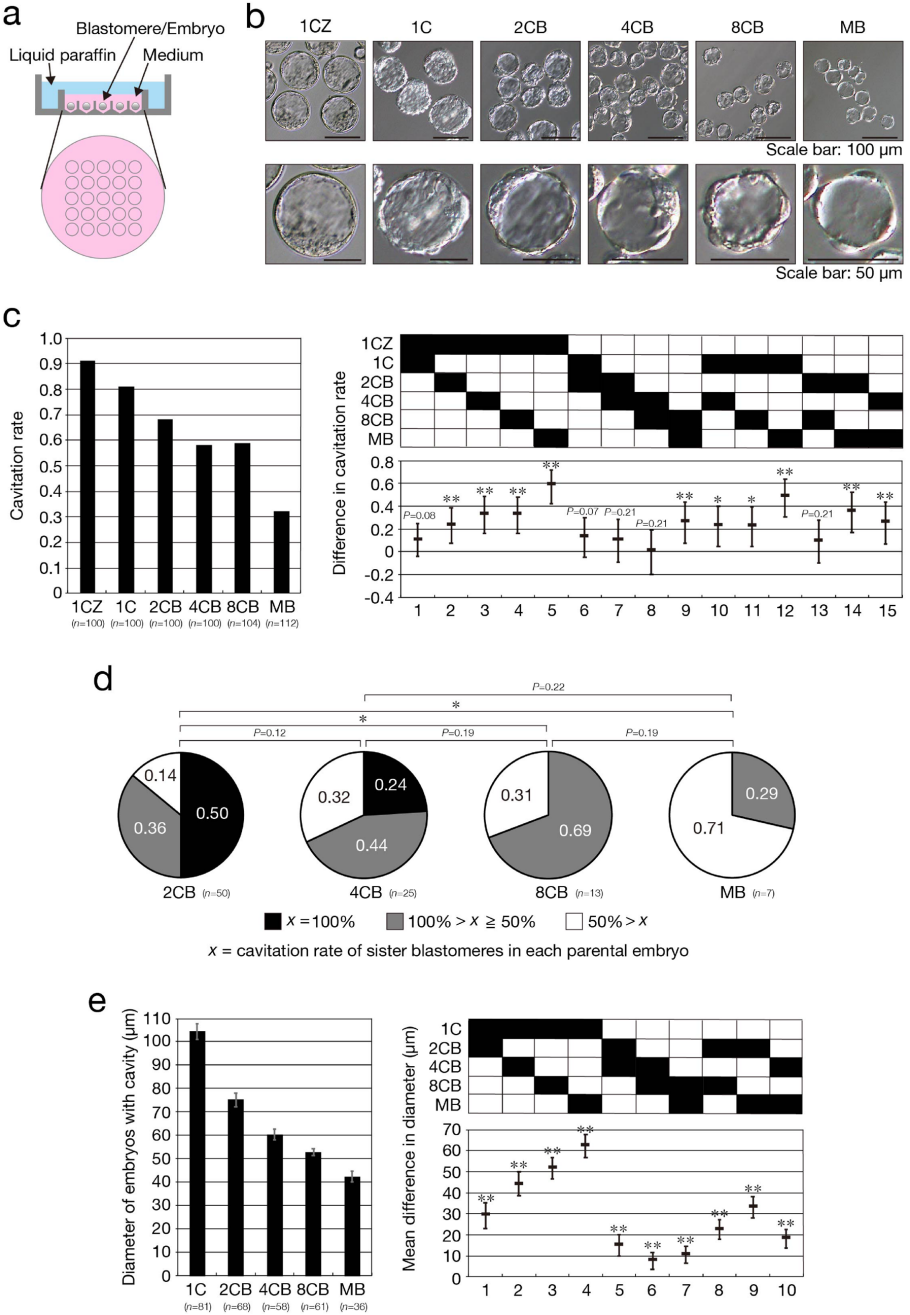
Reduced cavitation rate and size of embryos derived from single blastomeres. (**a**) Schematic representation of the in vitro culture system for isolated blastomeres. The image was created with the use of Illustrator version 24.0.1 (https://www.adobe.com/products/illustrator.html). (**b**) Representative bright-field images showing cavitation in embryos developed from single blastomeres. The lower panels are higher magnification views of embryos with a cavity in the upper panels. (**c**) Isolated blastomeres from embryos at the two-cell, four-cell, eight-cell, and morula stages as well as zygotes with or without the ZP were cultured for determination of the number of embryos with a cavity (> 50% by volume). The cavitation rate is shown in the left panel, and the difference in cavitation rates among the different types of embryo and its simultaneous 95% confidence interval (CI) are shown in the right panel. The *P* values were determined with the two-sided Fisher’s exact test followed by adjustment with the Benjamini-Hochberg (BH) method (false discovery rate [FDR] = 0.05). *P < 0.01, **P < 0.001. *n* indicates the number of zygotes or blastomeres. All data were obtained from three or four experiments performed on different days for each blastomere stage and were then analyzed. (**d**) Cavitation rate of sister blastomeres of individual parental embryos. The proportion of sister blastomeres that developed into an embryo with a cavity out of all sister blastomeres of each parental embryo in (**c**) is represented by *x*. Parental embryos were classified into three categories on the basis of the cavitation rate of their sister blastomeres. The *P* values were determined as in (**c**). *P < 0.01. *n* indicates the number of parental embryos. (**e**) Diameter of embryos with a cavity in (**c**). The overall mean values and 95% CIs are shown in the left panel, and the mean difference in diameters among the different types of embryo and its simultaneous 95% CI are shown in the right panel. Normality of the data was verified with the Jarque–Bera test (α = 0.05). The *P* values were determined by Welch’s test followed by the Games-Howell test. **P < 0.001. *n* indicates the number of embryos with a cavity.

To elucidate the distribution of totipotent cells in preimplantation mouse embryos, we cultured single isolated blastomeres in the wells of a culture plate (Fig. 1a). We refer to embryos developed from single blastomeres at the two-cell, four-cell, eight-cell, or morula stage or those developed from zygotes with or without the zona pellucida (ZP) as 2CB, 4CB, 8CB, MB, 1CZ, and 1C embryos, respectively. Early mammalian embryos develop into blastocysts before implantation, with blastocyst formation being accompanied by cavitation—formation of a fluid filled cavity known as the blastocyst cavity—as well as by segregation of the first two cell lineages^20^. We therefore first examined cavitation of 2CB, 4CB, 8CB, and MB embryos. For this examination, we used parental embryos from which all sister blastomeres were successfully isolated. 2CB (*n* = 100 from 50 parental embryos), 4CB (*n* = 100 from 25 parental embryos), 8CB (*n* = 104 from 13 parental embryos), and MB (*n* = 112 from 7 parental embryos) embryos were cultured in vitro (Fig. 1a) up to 96 h postinsemination (hpi). Cavitation began at the same time after insemination in 2CB, 4CB, 8CB, and MB embryos as in 1CZ and 1C embryos (Fig. 1b), suggesting that the preimplantation developmental clock of these former embryos is not compromised by blastomere isolation. On the other hand, the cavitation rate of embryos at 96 hpi was significantly decreased in 2CB (0.68), 4CB (0.58), and 8CB (0.59) embryos compared with 1CZ embryos (0.91), with this decrease being even more pronounced in MB embryos (0.32) (Fig. 1c). The cavitation rate of sister blastomeres of each parental embryo varied not only among parental embryos at different developmental stages but also among parental embryos at the same developmental stage, suggesting that blastomeres that possess the potential to form a cavity are not uniformly distributed in preimplantation embryos (Fig. 1d). Classification of the cavitation rate of sister blastomeres indicated that the proportion of parental embryos with high cavitation rates of sister blastomeres decreased as the development of the parental embryos progressed (Fig. 1d). In particular, parental embryos in which all sister blastomeres underwent cavitation were apparent at the two- and four-cell stages, but not at the eight-cell and morula stages, suggesting that concordance of the ability to form a cavity among all sister blastomeres is virtually absent at eight-cell and morula stages. Measurement of the diameter of embryos with a cavity revealed that the mean diameter of embryos derived from single blastomeres was significantly smaller than that of 1C embryos and decreased with developmental stage of the parental embryos (Fig. 1e). Together, these results suggested that the potential of single blastomeres to develop into embryos with a cavity as well as the diameter of such embryos decreases as the development of their parental embryos progresses.

**Figure 2:**
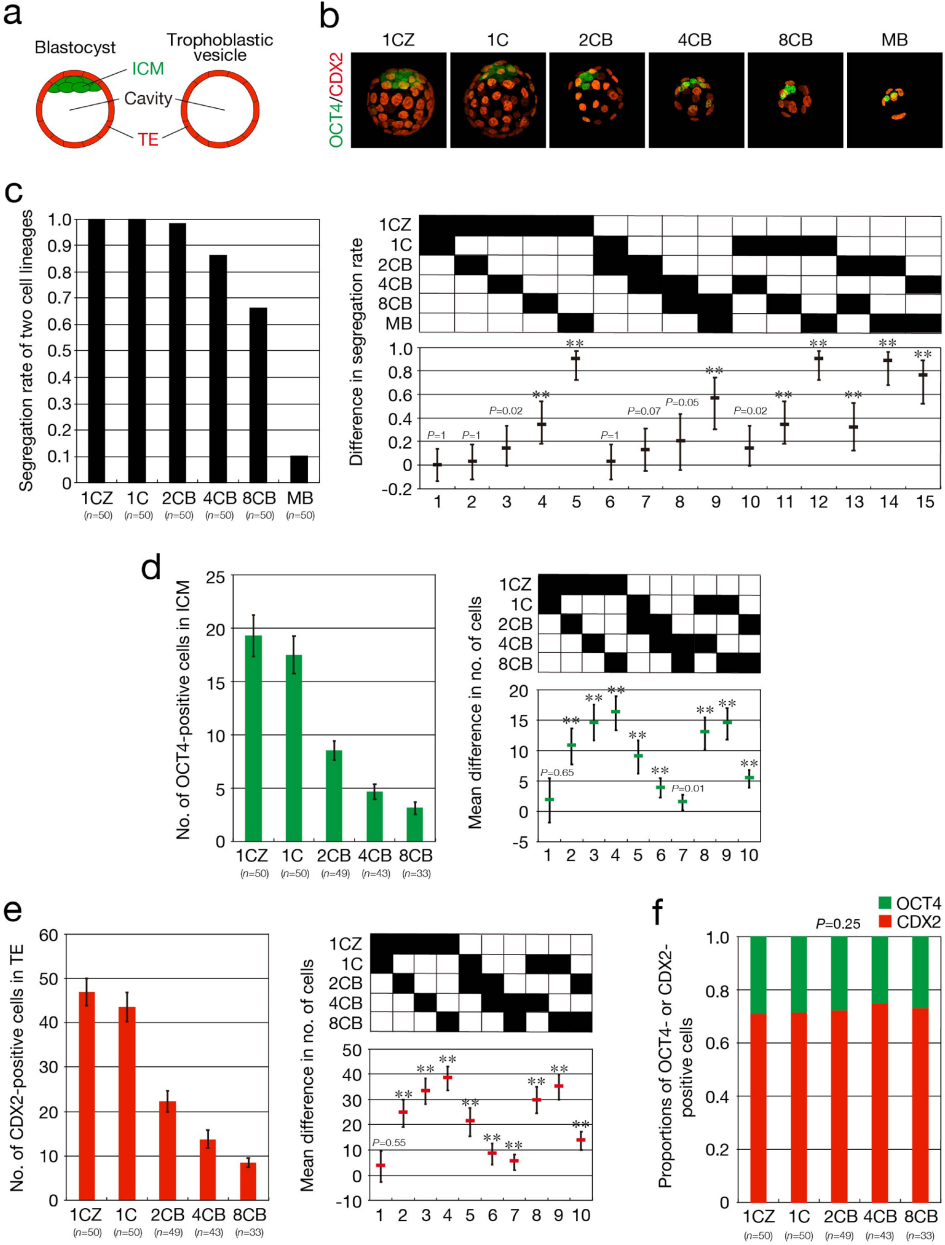
Reduced blastocyst formation and cell number for embryos derived from single blastomeres. (**a**) Schematic representation of a blastocyst and TV. The image was created with the use of Illustrator version 24.0.1 (https://www.adobe.com/products/illustrator.html). (**b**,**c**) 1CZ, 1C, 2CB, 4CB, 8CB, and MB embryos with a cavity at 96 hpi were subjected to immunofluorescence staining with anti-OCT4 and anti-CDX2 for determination of the segregation rate for the ICM and TE lineages. Representative images showing the mutually exclusive expression of OCT4 (green) and CDX2 (red) in the ICM and TE, respectively, are shown in (**b**). The segregation rate for the two cell lineages is shown in the left panel of (**c**), and the difference in segregation rates among the different types of embryo and its simultaneous 95% CI are shown in the right panel. The *P* values were determined with the two-sided Fisher’s exact test followed by adjustment with the BH method (FDR = 0.05). **P < 0.001. *n* indicates the number of embryos with a cavity. All data were obtained from three or four experiments performed on different days for each blastomere stage and were then analyzed. (**d**,**e**) The numbers of OCT4-positive cells in the ICM (**d**) and CDX2-positive cells in TE (**e**) of blastocysts in (**c**) were counted. The overall mean values and 95% CIs are shown in the left panels, and the mean difference in the numbers of OCT4-positive cells in the ICM or CDX2-positive cells in TE among the different types of embryo and its simultaneous 95% CI are shown in the right panels. Normality of the data was verified with the Jarque–Bera test (α = 0.05). The *P* values were determined by Welch’s test followed by the Games-Howell test. **P < 0.001. *n* indicates the number of blastocysts. (**f**) Proportions of OCT4-positive cells in the ICM and of CDX2-positive cells in TE of blastocysts in (**d**) and (**e**). The *P* value was determined with the two-sided Fisher’s exact test. *n* indicates the number of blastocysts.

We next investigated the segregation of the first two cell lineages—the ICM and TE—in embryos developed from single blastomeres^20^ (Fig. 2a). To this end, we examined expression of the transcription factors OCT4 and CDX2 in embryos with a cavity. OCT4 is expressed in cleavage-stage embryos and becomes restricted to the ICM after initiation of blastocyst formation^21^, whereas CDX2 is expressed specifically in TE at the blastocyst stage^22^. 2CB, 4CB, 8CB, and MB embryos were cultured in vitro up to 96 hpi, and embryos with a cavity (*n* = 50 each) were subjected to simultaneous immunofluorescence staining with antibodies to (anti-) OCT4 and anti-CDX2 (Fig. 2b). The segregation rate of the two cell lineages was significantly decreased in 4CB (0.86) and 8CB (0.66) embryos compared with 1CZ embryos (1.0), and this decrease was again most prominent in MB embryos (0.10) (Fig. 2c). All embryos in which mutually exclusive signals for OCT4 in the ICM and CDX2 in TE were not observed lacked only the signal for OCT4 in ICM, indicating that these embryos were most likely trophoblastic vesicles (TVs) in which all cells contribute to the wall of the vesicle and the ICM is completely absent^23^ (Fig. 2a). Determination of the number of cells in which the signal for OCT4 was prominent as well as that of cells in which the signal for CDX2 was prominent revealed that the mean numbers of both OCT4-positive cells in the ICM and CDX2-positive cells in TE of embryos derived from single blastomeres were significantly decreased compared with those for 1CZ blastocysts and that they declined with developmental stage of the parental embryos (Fig. 2d,e). In contrast, the proportions of OCT4-positive cells in the ICM and CDX2-positive cells in TE did not differ significantly between blastocysts derived from single blastomeres and 1CZ blastocysts (Fig. 2f). These results together suggested that the potential of single blastomeres to develop into blastocysts declines as the development of their parental embryos progresses and is virtually absent in blastomeres at the morula stage.

### Totipotent cells are present in preimplantation embryos up to the four-cell stage

**Figure 3:**
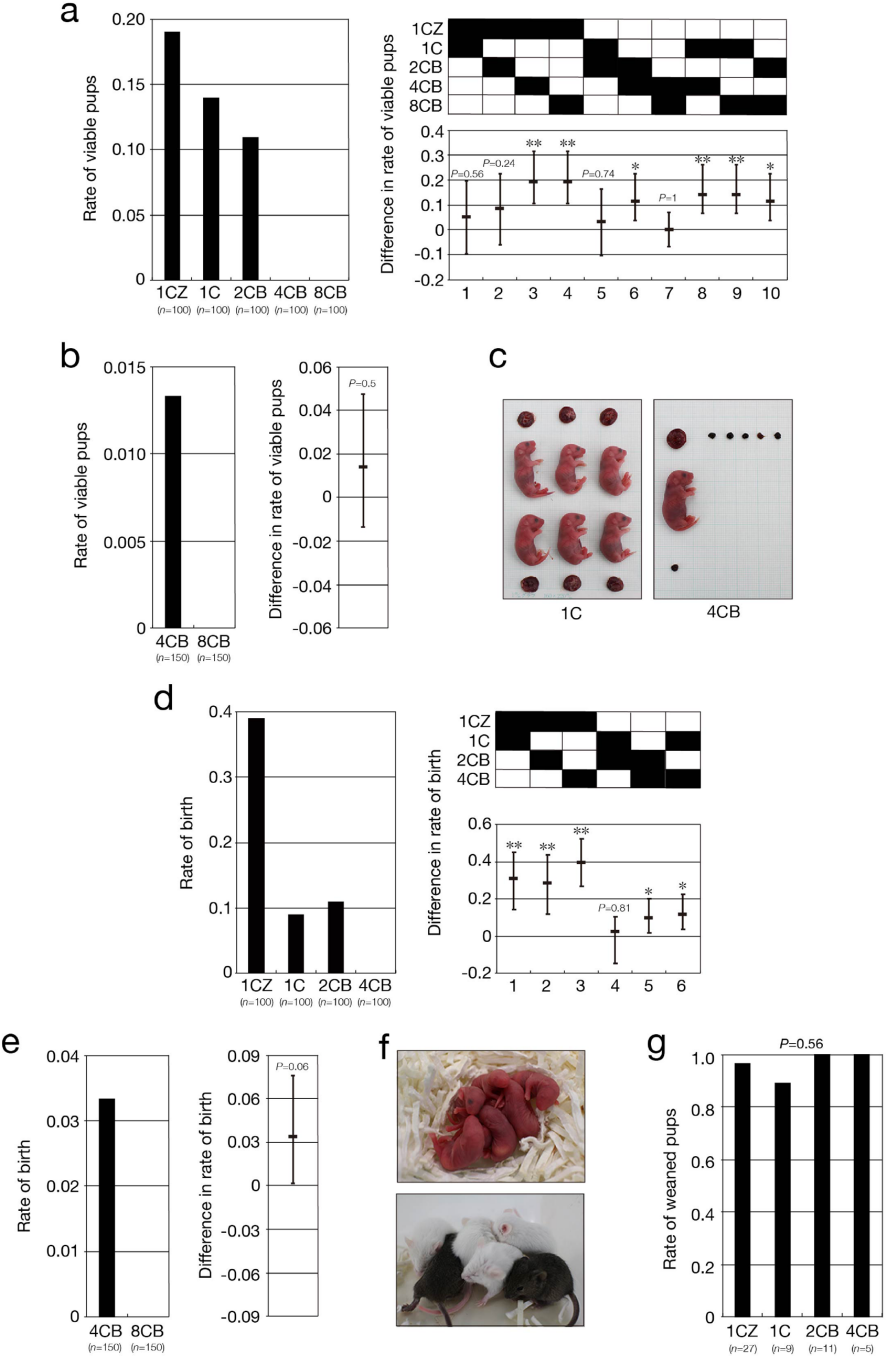
Totipotent cells distribute in preimplantation embryos up to the four-cell stage. (**a**,**b**,**d**,**e**) 1CZ, 1C, 2CB, 4CB, or 8CB pseudoblastocysts at 96 hpi were transferred surgically to the uterine horns of pseudopregnant (**a**,**b**) or pregnant (**d**,**e**) ICR female mice (*n* = 5 each) at a ratio of 20 (**a**,**d**) or 30 (**b**,**e**) pseudoblastocysts per mouse. Pregnancy status was determined by C-section at E18.5 (**a**,**b**) or after vaginal delivery (**d**,**e**). The rate of viable pups (or birth) was determined as the proportion of transferred pseudoblastocysts that gave rise to viable pups (left panels), and the difference in the rates of viable pups among the different types of embryo and its simultaneous 95% CI (**a**,**d**) or 95% CI (**b**,**e**) are shown in the right panels. The *P* values in (**a**) and (**d**) were determined with the two-sided Fisher’s exact test followed by adjustment with the BH method (FDR = 0.05), and those in (**b**) and (**e**) were determined with the two-sided Fisher’s exact test. *P < 0.01, **P < 0.001. *n* indicates the number of pseudoblastocysts transferred. Embryo transfer was performed on at least three different days for each blastomere stage. (**c**) Representative images of embryos, their placentas, and debris of absorbed embryos from a single litter derived from 1C or 4CB pseudoblastocysts at E18.5. (**f**) Representative images of neonatal mice (upper panel) and weaned mice (lower panel) derived from 4CB embryos. (**g**) Rate of 3-week-old weaned pups in (**d**) and (**e**). The *P* value was determined with the two-sided Fisher’s exact test. *n* indicates the number of neonatal mice.

Given that 2CB, 4CB, and 8CB embryos are sufficiently competent to develop into blastocysts, we evaluated their potential to develop to term in utero. For this evaluation, we used embryos with a cavity without assessing them for the presence of the ICM and refer to such embryos as pseudoblastocysts, given that it was often difficult to determine precisely whether embryos with a cavity contain the ICM by bright-field microscopy, especially for 4CB and 8CB embryos. On the other hand, we refer to embryos with a cavity in which the presence of the ICM was confirmed by immunostaining as blastocysts. Pseudoblastocysts thus include not only blastocysts but also TVs at the ratios indicated in Fig. 2c. 2CB, 4CB, and 8CB pseudoblastocysts that had been cultured in vitro up to 96 hpi (*n* = 100 each) were transferred surgically to the uterine horns of pseudopregnant foster female mice (*n* = 5 each, 20 pseudoblastocysts per mouse), and the frequency of viable pups or absorbed embryos was determined. Cesarean section (C-section) at embryonic day 18.5 (E18.5) revealed that viable pups were obtained from 2CB embryos but not from 4CB or 8CB embryos (Supplementary Table S1), consistent with the results of previous studies^12,23,24^. The rate of viable pups obtained from 1C (0.14, 14 pups obtained from 4 of 5 foster mice) or 2CB (0.11, 11 pups obtained from 5 of 5 foster mice) embryos did not differ significantly from that of those obtained from 1CZ embryos (0.19, 19 pups obtained from 4 of 5 foster mice) (Fig. 3a, Supplementary Fig. S1a). Implantation sites without viable embryos were observed in the uterine horns of pseudopregnant foster female mice in which 1CZ, 1C, and 2CB embryos were transferred, but not in those in which 4CB or 8CB embryos were transferred (Supplementary Table S1). The rate of absorbed embryos derived from 1C (0.13, 13 absorbed embryos for 4 of 5 foster mice) or 2CB (0.09, 9 absorbed embryos for 2 of 5 foster mice) embryos after implantation did not differ significantly from that of those derived from 1CZ embryos (0.20, 20 absorbed embryos for 4 of 5 foster mice) (Supplementary Fig. S1b,c). To examine further whether 4CB or 8CB embryos can develop to term, we increased the number of pseudoblastocysts transferred to pseudopregnant foster females to 30 per mouse (150 pseudoblastocysts transferred to 5 mice). In contrast to previous studies^23–25^, viable pups were obtained from 4CB embryos by C-section at E18.5 at a rate of 0.01 (2 pups obtained from 2 of 5 foster mice) (Fig. 3b,c; Supplementary Fig. S1d; Supplementary Table S2). Implantation sites of 4CB pseudoblastocysts without viable embryos were also observed at a rate of 0.05 (7 absorbed embryos for 2 of 5 foster mice) (Supplementary Fig. S1e,f; Supplementary Table S2). On the other hand, neither viable pups nor implantation sites were apparent for 8CB embryos (Fig. 3b,c; Supplementary Fig. S1d–f; Supplementary Table S2). The weight of pups or the placenta derived from 1C, 2CB, or 4CB embryos did not differ significantly from that of those derived from 1CZ embryos, with the exception that the placenta for 2CB embryos was slightly heavier than that for 1CZ embryos (Supplementary Fig. S1g,h).

Given that our results indicated that 2CB and 4CB embryos possess the ability to develop to term, we next examined whether neonates derived from such embryos develop into fertile adults. 2CB and 4CB pseudoblastocysts that had been cultured in vitro up to 96 hpi (*n* = 100 each) were transferred surgically to the uterine horns of pregnant foster female mice (*n* = 5 each, 20 pseudoblastocysts per mouse), and the rate of viable pups delivered through the vagina was determined. All foster female mice in which pseudoblastocysts had been transferred gave birth to pups derived from their own embryos and, in some instances, from the transferred embryos (Supplementary Table S3). Pups derived from transferred embryos (black eyes and a dark coat) were distinguished from the dam’s own pups (red eyes and a white coat) on the basis of eye and coat color. Viable pups were obtained from 2CB embryos but not from 4CB embryos (Supplementary Table S3). The rate of viable pups was significantly decreased for 1C (0.09, 9 pups obtained from 4 of 5 foster mice) and 2CB (0.11, 11 pups obtained from 4 of 5 foster mice) embryos compared with 1CZ embryos (0.39, 39 pups obtained from 5 of 5 foster mice), indicating that the absence of a ZP might impede embryonic development if embryos with or without a ZP coexist in the uterus of pregnant mice (Fig. 3d, Supplementary Fig. S2a). We therefore again increased the number of 4CB pseudoblastocysts transferred to pregnant foster female mice up to 30 per mouse (150 pseudoblastocysts, 5 foster females), and again viable pups were successfully obtained from 4CB embryos at a rate of 0.03 (5 pups obtained from 4 of 5 foster mice) but not from 8CB embryos (Fig. 3e,f; Supplementary Fig. S2b; Supplementary Table S4). These results thus provided strong support for the notion that 4CB embryos have the potential to develop to term. For examination of the growth of 2CB (*n* = 11) and 4CB (*n* = 5) neonatal mice, the animals were allowed to nurse for 21 days after birth and then weaned. The weaning rate of 2CB and 4CB pups did not differ significantly from that of 1CZ pups (Fig. 3f,g). The body weight and length of weaned 2CB or 4CB mice also did not differ significantly from those of 1CZ mice, with the exception that the body weight and body length of 2CB male mice were slightly greater (Supplementary Fig. S2c,d). All weaned pups developed into outwardly healthy 8-week-old adults. For examination of their fecundity, the adults were housed with 8-week-old B6D2F1 counterparts and observed over a 2-month period. The fecundity of 2CB or 4CB mice was found to be similar to that of 1CZ mice (Supplementary Table S5, Supplementary Fig. S2e). These results together suggested that totipotent cells exist in mouse preimplantation embryos up to the four-cell stage, although their proportion is relatively small at this latter stage. Given that the absence of the ZP did not impair embryonic development under our experimental conditions, with the exception of that in utero in pregnant foster female mice, we used 1C embryos as a control in subsequent experiments.

### Transcriptomic characteristics of pseudoblastocysts derived from single blastomeres

**Figure 4:**
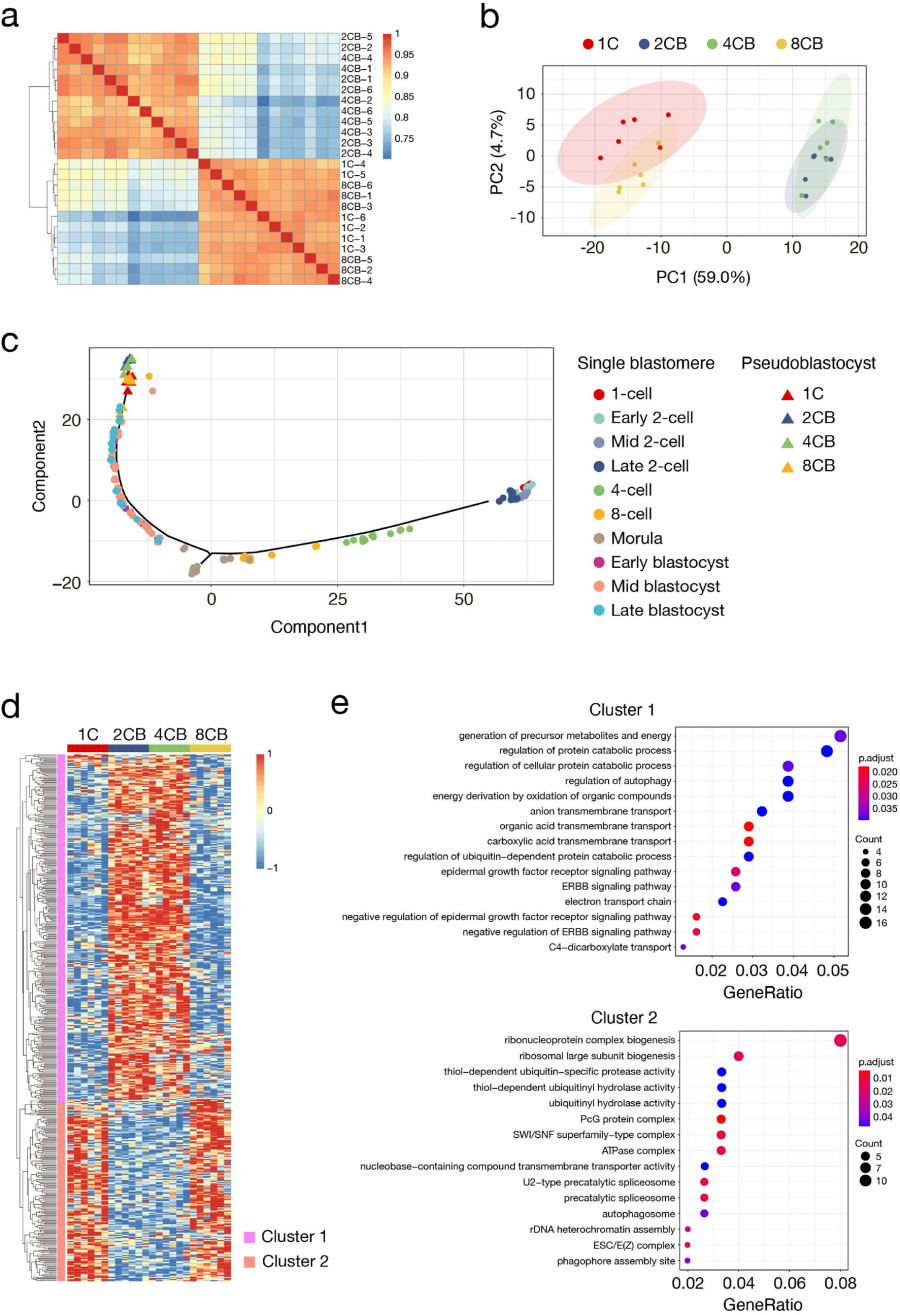
Transcriptomic analysis of pseudoblastocysts derived from single blastomeres. (**a**) Heat map for hierarchical clustering of Pearson’s correlations for 1C, 2CB, 4CB, and 8CB pseudoblastocysts (96 hpi) according to their gene expression profiles. Samples were clustered on the basis of their pairwise correlations. The color scale shows Pearson’s correlation coefficient. The image was created with the use of pheatmap version 1.0.12 (https://CRAN.R-project.org/package=pheatmap). All RNA-seq data were obtained by a single sequencing run and were then analyzed. (**b**) PCA analysis of the transcriptomes of 1C, 2CB, 4CB, and 8CB pseudoblastocysts (96 hpi). Shaded ellipses represent the 95% CI for blastomere stage. The image was created with the use of ggplot2 version 3.3.2^72^ (https://ggplot2.tidyverse.org). (**c**) Pseudotime trajectory analysis for the transcriptomes of 1C, 2CB, 4CB, and 8CB pseudoblastocysts (96 hpi) and for those of single blastomeres of preimplantation embryos at the indicated stages^27^ conducted with the use of Monocle2^73^. The image was created with the use of ggplot2 version 3.3.2^72^ (https://ggplot2.tidyverse.org). (**d**) Heat map showing relative expression patterns for the top 500 genes with the largest variance among 1C, 2CB, 4CB, and 8CB pseudoblastocysts (96 hpi) identified by DESeq2 (*P* < 0.01). The color scale shows the *z*-score. The image was created with the use of pheatmap version 1.0.12 (https://CRAN.R-project.org/package=pheatmap). (**e**) GO terms enriched in clusters 1 or 2 in (**d**). GO analysis was performed with the use of clusterProfiler version 3.10.0^74^. Count represents the number of differential genes related to the GO term. p.adjust represents the adjusted *P* value. GeneRatio represents the number of genes related to the GO term among the differentially expressed genes as a proportion of the total number of differentially expressed genes. The image was created with the use of clusterProfiler verion. 3.10.0^74^ (https://guangchuangyu.github.io/software/clusterProfiler/).

To characterize the molecular features of embryos derived from single blastomeres, we analyzed their transcriptomes. Each of six 1C, 2CB, 4CB, or 8CB pseudoblastocysts that had been cultured in vitro up to 96 hpi was thus subjected to single-embryo RNA sequencing (RNA-seq). Analysis of RNA-seq data by unsupervised hierarchical clustering revealed that the transcriptomes of 1C, 2CB, 4CB, and 8CB pseudoblastocysts segregated into two groups: one containing 1C and 8CB pseudoblastocysts, and the other containing 2CB and 4CB pseudoblastocysts (Fig. 4a). A similar segregation pattern of two groups dependent on global gene expression profile was also obtained by principal component analysis (PCA) (Fig. 4b), suggesting that the transcriptomic characteristics of 1C, 2CB, 4CB, and 8CB pseudoblastocysts are not directly related to their developmental potential. Given that major zygotic genome activation (ZGA) peaks between the two- and four-cell stages^26^, isolation of blastomeres at these stages might induce a delay in gene expression that results in a mismatch with morphological developmental stage of single-blastomere progeny. To examine this possibility, we performed pseudotime trajectory analysis of our RNA-seq data and a public RNA-seq data set for single blastomeres of mouse preimplantation embryos^27^. This analysis showed that global gene expression patterns of 2CB, 4CB, and 8CB pseudoblastocysts are similar to those of corresponding blastomeres in the preimplantation developmental sequence (Fig. 4c), eliminating the possibility that the difference in gene expression pattern between the two groups of pseudoblastocysts is attributable to a delay in gene expression.

We next examined differentially expressed genes among 1C, 2CB, 4CB, and 8CB pseudoblastocysts. Unsupervised hierarchical clustering analysis of differentially expressed genes identified two clusters in which gene expression patterns were clearly divided into two groups as observed in Fig. 4a,b (Fig. 4d). Gene ontology (GO) term enrichment analysis of the two clusters revealed that 1C and 8CB pseudoblastocysts were enriched in genes related to ribonucleoprotein biogenesis, ubiquitin-related processes, chromatin regulation, and spliceosomes, whereas 2CB and 4CB pseudoblastocysts preferentially featured genes related to protein catabolism, transmembrane transport, and epidermal growth factor and ERBB signaling pathways (Fig. 4e). These results suggested that isolation of blastomeres from embryos at the two-cell or four-cell stage disrupts their transcriptomes.

### Peri-implantation developmental potential is virtually absent in 8CB embryos

**Figure 5:**
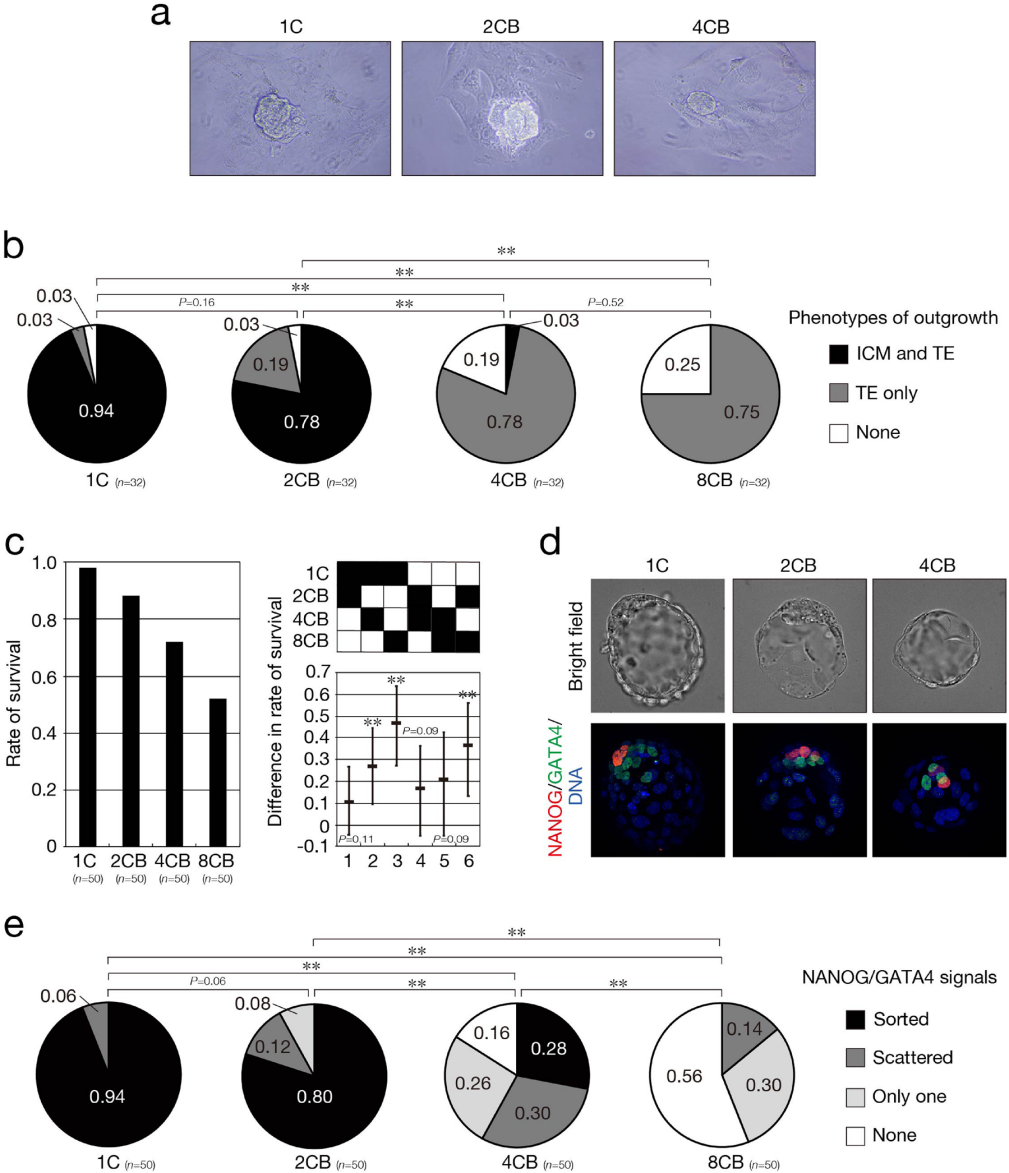
Defects in the ICM lineage of embryos derived from single blastomeres. (**a**) Representative phase-contrast images showing the outgrowth of pseudoblastocysts derived from single blastomeres after 5 days in a blastocyst outgrowth assay. (**b**) Phenotypes of 1C, 2CB, 4CB, and 8CB pseudoblastocysts in the blastocyst outgrowth assay were classified into three categories, and their proportions are shown. The *P* values were determined with the two-sided Fisher’s exact test followed by adjustment with the BH method (FDR = 0.05). **P < 0.001. *n* indicates the number of pseudoblastocysts. All data were obtained from four replicate experiments. (**c**) 1C, 2CB, 4CB, and 8CB pseudoblastocysts that had been cultured in vitro up to 96 hpi were then cultured further for determination of their survival rate at 120 hpi (judged on the basis of a maintained cavity). The rate of survival is shown in the left panel, and the difference in survival rates among the different types of pseudoblastocyst and its simultaneous 95% CI are shown in the right panel. The *P* values were determined as in (**b**). **P < 0.001. *n* indicates the number of pseudoblastocysts. All data were obtained from three or four experiments performed on different days for each blastomere stage and were then analyzed. (**d**,**e**) 1C, 2CB, 4CB, and 8CB pseudoblastocysts cultured in vitro up to 120 hpi were subjected to bright-field imaging and to immunofluorescence staining with anti-NANOG and anti-GATA4 for evaluation of EPI and PrE formation. Representative images demonstrating the mutually exclusive expression of NANOG (red) and GATA4 (green) in two distinct compartments corresponding to EPI and PrE, respectively, of 1C, 2CB, and 4CB blastocysts are shown in (**d**). DNA was stained with Hoechst 33342 (blue). The phenotypes of pseudoblastocysts with regard to formation of EPI and PrE were classified into four categories, and their proportions are shown in (**e**). Embryos were obtained by in vitro fertilization on at least three different days. The *P* values were determined as in (**b**). **P < 0.001. *n* indicates the number of pseudoblastocysts.

Given that the differences in transcriptomes among 1C, 2CB, 4CB, and 8CB pseudoblastocysts did not appear to be correlated with those in their developmental potential, we next evaluated the peri-implantation developmental potential of 2CB, 4CB, and 8CB embryos. We thus performed a blastocyst outgrowth assay that shows some analogy to the implantation process^28,29^. 2CB, 4CB, and 8CB pseudoblastocysts that had been cultured in vitro up to 96 hpi (*n* = 32 each) were transferred to plastic tissue culture plates that had been coated with gelatin, and their outgrowth was monitored over 5 days. In the outgrowth process, blastocysts attach to the plastic plates, TE cells begin to migrate out of the tissue within 3 days, and expansion of the ICM surrounded by trophoblast giant cells that have differentiated from TE cells is apparent after 5 days (Fig. 5a). The proportion of 4CB pseudoblastocysts (0.03) manifesting this normal phenotype was significantly smaller than that of 1C (0.94) or 2CB (0.78) pseudoblastocysts, and the phenotype was not observed at all for 8CB pseudoblastocysts (Fig. 5b). In contrast, the proportion of pseudoblastocysts showing phenotypes characterized by outgrowth of TE only without ICM or by outgrowth of neither TE nor ICM was significantly increased for 4CB and 8CB, suggestive of a severe defect in the ICM lineage rather than in the TE lineage in 4CB and 8CB embryos.

We next investigated the formation of EPI and PrE in the ICM. Formation of EPI and PrE is a multistep process that consists of specification of the corresponding lineages, maturation of the lineages, and cell sorting that gives rise to two distinct compartments, an EPI cluster and a PrE epithelium. In the lineage specification process, the expression of marker genes of PrE and EPI becomes restricted in a mutually exclusive pattern to the corresponding precursors, which appear scattered in the ICM. Expression of *Nanog* thus becomes restricted to EPI precursors, whereas PrE precursors at this stage begin to express *Gata4*, with expression of such markers being followed by lineage maturation and establishment of the specific gene networks that underlie the differentiation of each lineage. PrE and EPI then become spatially segregated into two distinct compartments: PrE cells eventually congregate on the surface of the ICM exposed to the blastocyst cavity and mature to form a polarized epithelial layer, whereas EPI becomes enclosed between PrE and TE^30–33^. 2CB, 4CB, and 8CB pseudoblastocysts that had been cultured in vitro up to 96 hpi (*n* = 50 each) were cultured further up to 120 hpi for examination of the formation of EPI and PrE in the ICM. The rate of survival during this extended culture period was significantly decreased for 4CB (0.72) and 8CB (0.52) pseudoblastocysts compared with 1C pseudoblastocysts (0.98) (Fig. 5c). Pseudoblastocysts at 120 hpi (*n* = 50 each) were then subjected to simultaneous staining with anti-NANOG and anti-GATA4 (Fig. 5d). The proportion of pseudoblastocysts in which mutually exclusive scattered signals for NANOG and GATA4 were observed in the ICM or in which sorted signals for each protein were observed in distinct compartments was also significantly decreased for 4CB (0.58) and 8CB (0.14) relative to 1C (1.0) and 2CB (0.92), suggesting that the specification of EPI and PrE lineages is severely defective in 4CB and 8CB embryos (Fig. 5e). Furthermore, the proportion of embryos with sorted signals for NANOG and GATA4 in distinct compartments was also significantly decreased for 4CB (0.28) compared with 1C (0.94) and 2CB (0.80), with no such sorting being detected at all for 8CB (Fig. 5e), suggesting that the cell sorting that underlies the formation of two distinct compartments is also defective in 4CB and 8CB embryos. Pseudoblastocysts in which the signal for neither NANOG nor GATA4 was observed were most likely TVs.

**Figure 6:**
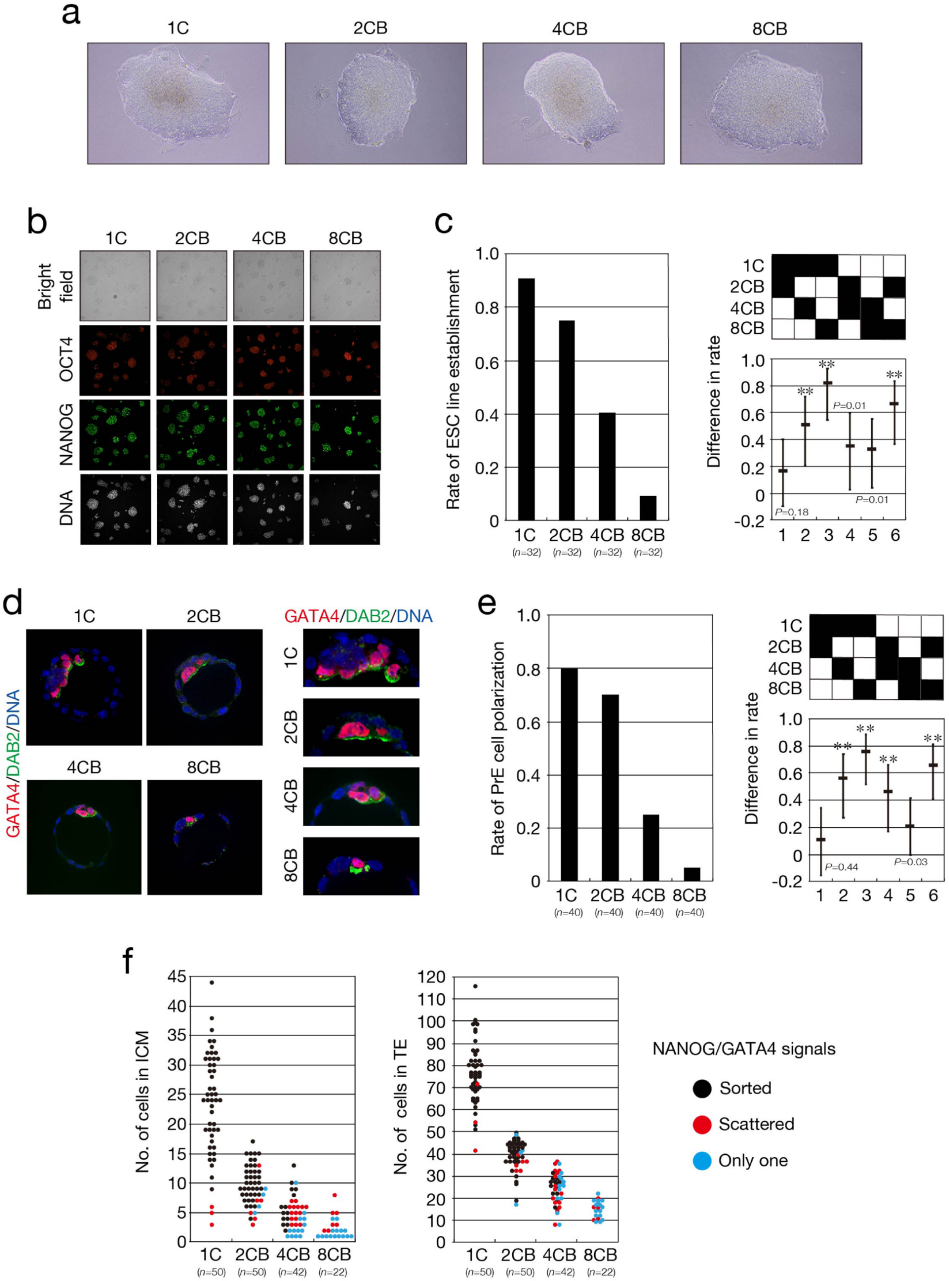
Impaired formation of EPI and PrE in embryos derived from single blastomeres. (**a**–**c**) 1C, 2CB, 4CB, and 8CB pseudoblastocysts at 96 hpi were cultured for the establishment of ESC lines, which were subjected to immunofluorescence staining with anti-OCT4 and anti-NANOG for determination of their pluripotency. Representative phase-contrast images showing ICM outgrowth of pseudoblastocysts after culture for 14 days are shown in (**a**). Representative bright-field as well as OCT4 (red) and NANOG (green) immunofluorescence images of established ESC lines are shown in (**b**). DNA was stained with RedDot2 (white). The rate of ESC line establishment is shown in the left panel of (**c**), and the difference in the rates among the different types of pseudoblastocyst and its simultaneous 95% CI are shown in the right panel. The *P* values were determined with the two-sided Fisher’s exact test followed by adjustment with the BH method (FDR = 0.05). **P < 0.001. *n* indicates the number of pseudoblastocysts. All data were obtained from four replicate experiments. (**d**,**e**) 1C, 2CB, 4CB, and 8CB pseudoblastocysts at 120 hpi were stained with anti-GATA4 and anti-DAB2 for determination of the rate of PrE polarization. Representative images showing the expression of GATA4 (red) and apical-side expression of DAB2 (green) in cells located in the PrE compartment of blastocysts are shown in (**d**). DNA was stained with Hoechst 33342 (blue). The right panels are higher magnification views of the ICM of embryos in the left panels. The rate of PrE polarization is shown in the left panel of (**e**), and the difference in the rates of PrE polarization among the different types of embryo together with its simultaneous 95% CI are shown in the right panel. The *P* values were determined as in (**c**). **P < 0.001. *n* indicates the number of pseudoblastocysts. All data were obtained from three or four experiments performed on different days for each blastomere stage and were then analyzed. (**f**) Number of cells in the ICM (left panel) and in TE (right panel) of the blastocysts in Fig. 5e.

We next examined the maturation of functional EPI. To this end, we established ESC lines from progenitor cells that reside in EPI^34^. 2CB, 4CB, and 8CB pseudoblastocysts that had been cultured in vitro up to 96 hpi (*n* = 32 each) were transferred to plastic tissue culture plates that had been coated with gelatin and were cultured under the 2i/LIF (leukemia inhibitory factor) condition^35^. ESC lines were established by disaggregation of the ICM outgrowth followed by six passages of the dissociated cells, and their pluripotency was assessed on the basis of expression of the markers OCT4 and NANOG (Fig. 6a,b). The rate of establishment of ESC lines was significantly decreased for 4CB (0.41, 13 lines) and 8CB (0.09, 3 lines) pseudoblastocysts compared with 1C (0.91, 29 lines) and 2CB (0.75, 24 lines) pseudoblastocysts (Fig. 6c), suggesting that differentiation of the EPI lineage from ICM is defective in 4CB and 8CB pseudoblastocysts. We then investigated maturation of PrE. Congregated PrE on the surface of the ICM matures to form an epithelial layer. The epithelialization of PrE begins with its polarization, the first sign of which is the apical localization of DAB2^31,33,36^. To examine the polarization of PrE, we subjected 2CB, 4CB, and 8CB pseudoblastocysts that had been cultured in vitro up to 120 hpi (*n* = 40 each) to simultaneous staining with anti-DAB2 and anti-GATA4 (Fig. 6d). The proportion of embryos in which both the signal for GATA4 and apical localization of the signal for DAB2 were apparent in cells on the surface of the ICM exposed to the blastocyst cavity was significantly reduced for 4CB (0.25) and 8CB (0.05) compared with 1C (0.8) and 2CB (0.7) (Fig. 6e), suggesting that differentiation of the PrE lineage from ICM is also markedly impaired in 4CB and 8CB pseudoblastocysts.

Given that the formation of EPI and PrE may be regulated in a non–cell autonomous manner by a combination of paracrine signaling and cell–cell interaction^31,33,37^, it might be expected to require a sufficient number of cells in the ICM. To examine this possibility, we determined the number of cells in the ICM of pseudoblastocysts at 120 hpi that were positive for staining with anti-NANOG or anti-GATA4 in Fig. 5e. Most embryos that possessed eight or more cells in the ICM manifested sorted signals for GATA4 and NANOG in two distinct compartments (Fig. 6f). In contrast, most embryos that manifested scattered or only one of the signals for NANOG and GATA4 possessed fewer than eight and five cells in the ICM, respectively (Fig. 6f), indicating that the number of cells in the ICM tends to correlate with the formation of EPI and PrE. In contrast, such a correlation was not observed for the number of cells in TE (Fig. 6f). These results thus suggested that the peri-implantation developmental potential of single blastomeres declines as the development of their parental embryos progresses and is virtually absent in those isolated from eight-cell embryos.

### Concordance of the ability to form EPI and PrE among sister blastomeres is essentially absent in embryos at the four-cell stage

**Figure 7:**
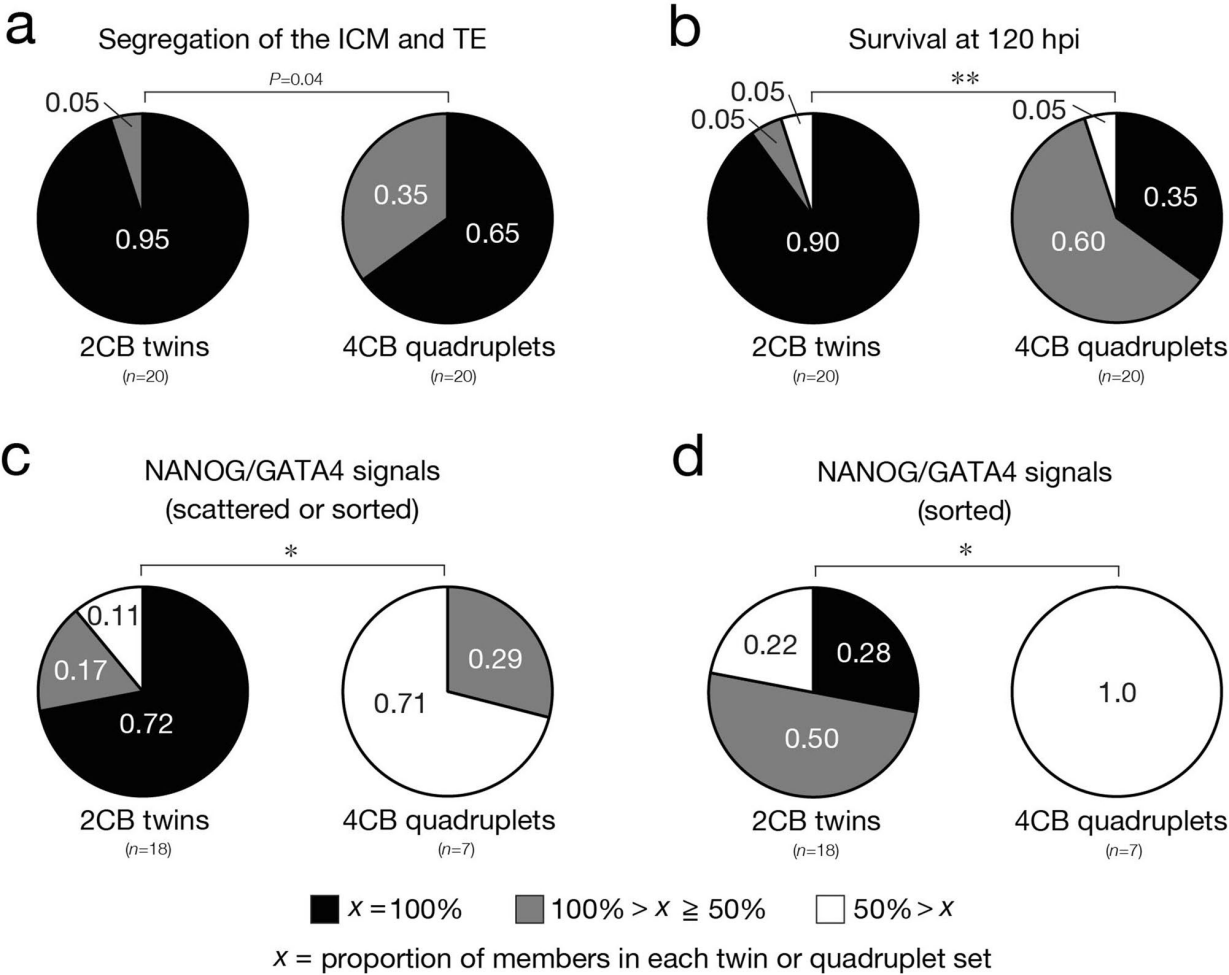
Discordance in developmental ability for isolated single sister blastomeres of the same parental embryos. (**a**) 2CB and 4CB pseudoblastocysts (96 hpi) derived from twin or quadruplet blastomeres, respectively, of parental embryos were subjected to immunofluorescence staining with anti-OCT4 and anti-CDX2 for determination of the segregation rate for the ICM and TE lineages. The proportion of pseudoblastocysts in which the ICM and TE were segregated out of all pseudoblastocysts derived from each twin or quadruplet set of blastomeres is represented by *x*. Parental embryos were classified into three categories on the basis of this proportion. The *P* value was determined with the two-sided Fisher’s exact test. *n* indicates the number of twin or quadruplet blastomere sets (parental embryos). All data were obtained from three or four experiments performed on different days for each blastomere stage and were then analyzed. (**b**) 2CB and 4CB pseudoblastocysts that had been cultured in vitro up to 96 hpi were then cultured further for determination of their survival rate at 120 hpi (judged on the basis of a maintained cavity). The proportion of pseudoblastocysts that survived during this extended culture period out of all pseudoblastocysts derived from each twin or quadruplet set of blastomeres is represented by *x*. Parental embryos were classified into three categories on the basis of this proportion. **P < 0.001, determined as in (**a**). *n* indicates the number of twin or quadruplet blastomere sets (parental embryos). All data were obtained from three or four experiments performed on different days for each blastomere stage and were then analyzed. (**c**,**d**) 2CB and 4CB pseudoblastocysts cultured in vitro up to 120 hpi in (**b**) were subjected to immunofluorescence staining with anti-NANOG and anti-GATA4 for evaluation of EPI and PrE formation. The phenotypes of pseudoblastocysts with regard to formation of EPI and PrE were classified into four categories as shown in Fig. 5e. The proportion of pseudoblastocysts that showed mutually exclusive scattered or sorted signals for NANOG and GATA4 (**c**) or sorted signals for these proteins (**d**) in the ICM out of all pseudoblastocysts derived from each twin or quadruplet set of blastomeres is represented by *x*. Parental embryos were classified into three categories on the basis of these proportions. *P < 0.01, determined as in (**a**). *n* indicates the number of twin or quadruplet blastomere sets (parental embryos).

Given that 2CB and 4CB pseudoblastocysts are sufficiently competent to form EPI and PrE, we investigated interblastomere differences of the parental two-cell and four-cell embryos in cell lineage specification associated with pre- and peri-implantation development. To this end, we evaluated the potential for lineage specification in sister blastomeres that gave rise to 2CB or 4CB pseudoblastocysts (*n* = 20 twin or quadruplet sets of blastomeres). We first investigated the segregation of ICM and TE in the same manner as in Fig. 2c. Classification of twin and quadruplet sets of blastomeres according to the ICM/TE segregation rate of the corresponding 2CB or 4CB pseudoblastocysts revealed that the proportion of twins or quadruplets in which all members formed the ICM and TE was 0.95 and 0.65, respectively, indicating that all sister blastomeres have a concordant ability to form the ICM and TE in 48% (rate of cavitation [0.5] multiplied by the rate of ICM/TE segregation [0.95]) of two-cell embryos and 16% (rate of cavitation [0.24] multiplied by the rate of ICM/TE segregation [0.65]) of four-cell embryos (Fig. 1d, Fig. 7a). We next investigated the formation of EPI and PrE in the same manner as in Fig. 5c,e. Classification of twin and quadruplet sets of blastomeres according to the survival rate of the corresponding 2CB or 4CB pseudoblastocysts during the additional 24-h culture period revealed that the proportion of twins or quadruplets in which all members survived was 0.90 and 0.35, respectively (Fig. 7b). In the case of parental embryos for which all blastomere-derived pseudoblastocysts survived (*n* = 18 twin or 7 quadruplet sets), the proportion of twins or quadruplets in which all members showed either mutually exclusive scattered signals for NANOG and GATA4 in the ICM or sorted signals for each protein was 0.72 (twins) and 0 (quadruplets), respectively (Fig. 7c). Similar results were obtained for the proportion of twins or quadruplets in which all members showed signals for NANOG in the ICM, indicating that all sister blastomeres have a concordant ability to form presumptively functional EPI in 32% (rate of cavitation [0.5] multiplied by the survival rate [0.90] and the NANOG signal rate [0.72]) of two-cell embryos but that such an ability is essentially absent at the four-cell stage (Supplementary Fig. S3). Furthermore, the proportion of twin sets in which both members showed sorted signals for NANOG and GATA4 in distinct compartments was only 0.28, indicating that both sister blastomeres have a concordant ability to form EPI and PrE in 13% (rate of cavitation [0.5] multiplied by the survival rate [0.90] and the rate of sorted signals for NANOG and GATA4 in distinct compartments [0.28]) of two-cell embryos (Fig. 7d). These results thus suggested that concordance of the ability to form EPI and PrE among all sister blastomeres declines as the development of their parental embryos progresses and is virtually absent in embryos at the four-cell stage.

## Discussion

We have here shown that totipotency of mouse zygotes extends to blastomeres at the four-cell stage, although the proportion of totipotent blastomeres is relatively small at the four-cell stage, and that pre- and peri-implantation developmental potential are essentially lost in blastomeres of embryos at the morula and eight-cell stages, respectively. Characterization of the molecular features of 2CB, 4CB, and 8CB pseudoblastocysts indicated that isolation of single blastomeres during ZGA results in disturbance of the transcriptome in progeny cells. Our data further show that concordance of peri-implantation developmental potential among all sister blastomeres of the same embryo is relatively low or essentially lost in those at the two-cell and four-cell stages, respectively, suggesting that the developmental potential of single blastomeres is no longer uniform after the first cell division.

Zygotes and blastomeres at the two-cell stage have been thought to be the only mouse cells that are totipotent. This notion was based on experimental evidence that blastomeres at the two-cell stage give rise to fertile adults when placed in a uterus, but that embryos derived from those at either the four- or eight-cell stage do not develop to term^12,23–25^. However, our experimental evidence now suggests that blastomeres at the four-cell stage are capable of giving rise to a fully formed individual. We have shown that transplantation of 4CB pseudoblastocysts that had been cultured in vitro into pseudopregnant or pregnant foster female mice resulted in successful development of embryos to term at a rate of 1.3% and 3.3%, respectively, and that 4CB neonates developed into outwardly healthy fertile adults. This discrepancy between the results of our study and those of three previous studies^23–25^ may be attributable to differences in experimental conditions. First, we transferred 150 pseudoblastocysts into foster female mice when we obtained the viable 4CB pups, whereas the number of embryos transferred into pseudopregnant foster female mice was 25 (single blastomeres)^23^ or 20 (blastocysts)^24^ in two of the three previous studies, with the number of blastocysts transferred not being indicated in the remaining study^25^. Given that the rate of development to term was 1.3% for 4CB pseudoblastocysts in pseudopregnant mice under our experimental conditions, the number of transferred embryos in the previous studies was likely too small to obtain viable pups. Second, we transferred pseudoblastocysts that had been cultured in vitro on the round bottom of tiny wells (280 μm in diameter and 160 μm in depth) of a cell culture dish, whereas blastocysts that had been cultured in vitro on the flat bottom of a cell culture dish^24^ or microplatform^25^ were transferred in two of the three previous studies. Embryonic development may be affected by the accumulation of paracrine and autocrine factors. Simulation of diffusion in the cell culture dish adopted for our experiments indicated that biological macromolecules secreted by embryos may tend to stay inside the corresponding well or move to neighboring wells, thereby facilitating autocrine and paracrine effects^38^. The rate of development of 2CB and 4CB embryos into blastocysts and their quality has been shown to be enhanced with the use of a microplatform compared with conventional microdroplet culture^25^, possibly as a result of the accumulation of paracrine and autocrine factors promoted by the associated reduction in the volume of culture medium. Continuous medium perfusion in conventional microdroplet culture on the flat bottom of a cell culture dish might deprive blastomeres during preimplantation development of paracrine and autocrine factors that promote their further development. Furthermore, physical restriction of available space during culture might affect cell association within embryos that influences cell differentiation and subsequent embryonic development to term. For example, four main types of conformation based on the total number of cell-to-cell contacts made within each embryo were observed for 1C or 2CB embryos at the four-cell stage that had been cultured in vitro without the ZP^39,40^. 1C embryos at the four-cell stage with the lowest number of cell–cell contacts gave rise to more blastocysts with a small ICM and poor survival after transfer to foster females than did those with the highest number of cell–cell contacts, although the in vitro development of embryos into blastocysts did not differ significantly among the four types of conformation^39^. Culture of single blastomeres without the ZP on the flat bottom of a cell culture dish or microplatform might increase the proportion of embryos that survive poorly after transfer. Third, blastocysts cultured with inhibitors of mitogen-activated protein kinase kinase (MEK) and glycogen synthase kinase 3 (GSK3) signaling^24^, or with LIF and inhibitors of MEK and transforming growth factor–β (TGF-β)^25^, were transferred in two of the three previous studies. Given that inhibition of extracellular signal–regulated kinase (ERK) signaling does not impede blastocyst formation but suppresses development of PrE^41^, culture of 4CB blastocysts in the presence of a MEK inhibitor might result in a deficiency of PrE cells required for further development, although these various culture conditions were found to increase the number of cells in ICM^42^ or the number of EPI cells^24^ compared with control embryos. In addition to the increased number of embryos transferred, the low birth rate and absence of the ZP for transferred embryos in our experiments raise the possibility that live pups were the product of fusion between two pseudoblastocysts in the uterus. However, evidence that embryos after the 16-cell stage lose the ability to fuse with each other might argue against this possibility^43,44^. Our findings thus refine current knowledge of totipotency in mouse cells.

The mechanism that underlies the loss of totipotency in blastomeres remains largely elusive. However, the phenotypes of blastomeres at pre- and peri-implantation stages of development may shed light on this mechanism. Before implantation, embryos develop into blastocysts characterized by cavitation and segregation of ICM and TE lineages. Single blastomeres have been thought to possess preimplantation developmental potential on the basis of the observation that blastomeres at the two-, four-, and eight-cell stages are capable of developing into embryos with a cavity^12,45,46^. We have now shown that 2CB, 4CB, and 8CB embryos are able to develop into blastocysts in which cavitation as well as bifurcation of ICM and TE lineages occur. However, we found that this potential is virtually absent in MB embryos. Only 3% (rate of cavitation [0.3] multiplied by the rate of lineage segregation [0.1]) of examined MB embryos developed into blastocysts in vitro. This developmental phenotype of MB embryos might be attributable to TE lineage specification in morula-stage embryos. Formation of the blastocyst cavity is a morphological feature of TE maturation^47^. Sixteen-cell morula-stage embryos comprise two types of cell: outer cells and inner cells that remain on the outside of embryos or have become localized to the inside, respectively^48^. Given that outer cells are presumptive TE, they are able to produce mature TE, whereas inner cells might not^47^. Thus, only embryos developed from single outer cells may form the cavity. However, outer cells may not be able to generate ICM lineage cells as a result of their TE lineage commitment. Most embryos with a cavity derived from outer cells might therefore lack the ICM. Although dissociation followed by reaggregation of 16-cell morula-stage embryos has shown that both outer and inner cells remain competent to produce both ICM and TE cells^49,50^, this competence may require cell–cell interactions, with the result that isolated single blastomeres from morula-stage embryos may not be so competent. Our data collectively support the notion that totipotency of zygotes does not extend to single blastomeres of morula-stage embryos.

Peri-implantation development is critical for mammalian embryogenesis. During this time, connections between embryonic and maternal tissue are set up. Embryonic development after the blastocyst stage requires implantation into the uterine endometrium. However, a process that shows similarities to implantation can be achieved in vitro with the blastocyst outgrowth assay. With the use of this assay, we found that the developmental potential of the ICM lineage, rather than that of the TE lineage, is markedly defective in 4CB and 8CB embryos, consistent with the previous observation that most decidua of such embryos contain only a few trophoblast giant cells at 5.5 days postcoitus (dpc)^23^. During the peri-implantation period, EPI and PrE are formed from the ICM of the blastocyst in a multistep process. This process begins with specification of the EPI and PrE lineages, which is followed by their maturation and cell sorting to form two distinct compartments, an EPI cluster and a PrE epithelium. We have now shown that 2CB and 4CB blastocysts are able to develop into embryos in which EPI and PrE formation occurs. However, we found that this potential is greatly diminished and is virtually absent in 4CB and 8CB blastocysts, respectively. Our results thus suggest that impaired maturation of PrE in addition to defective formation of functional EPI cells might contribute to the marked reduction in the number of 4CB viable pups and lack of 8CB viable pups. Developmental failure of 4CB and 8CB embryos has previously been proposed to result from a small size of the ICM^12^, although the precise mechanism has remained elusive. A non–cell autonomous mechanism involving a combination of paracrine signaling and cell–cell interaction has been implicated in the formation of EPI and PrE, including lineage specification and maturation, cell sorting, and cell survival^31,33^. Given that such a non–cell autonomous mechanism might be expected to require an appropriate number of cells to operate, the formation of EPI and PrE might not be able to occur properly with only a limited number of cells. We have now shown that the number of cells in the ICM correlated with the progress of EPI and PrE formation, and that a minimum of eight cells in the ICM is required for reliable formation of these tissues, including their spatial segregation into two distinct compartments, thus supporting the previously proposed hypothesis. Formation of the ICM or EPI, or the number of the corresponding cells, is often adopted as a measure of the developmental potential of embryos. Our results suggest that formation of PrE is also an important indicator for proper evaluation of the potential of embryos to develop to term. Collectively, our data support the notion that totipotency of zygotes does not extend to single blastomeres of embryos at the eight-cell stage.

A unique feature of development before implantation is the presence of maternal RNAs and proteins in the embryo. Transcription from the newly formed zygotic genome during ZGA establishes the transcriptome that is essential for subsequent embryonic development. We found that the transcriptomes of 1C, 2CB, 4CB, and 8CB pseudoblastocysts could be classified into two groups, one group comprising 1C and 8CB and the other consisting of 2CB and 4CB. The differences in transcriptomes between these two groups might be attributable to the isolation of blastomeres during ZGA in the case of 2CB and 4CB embryos and may lead to developmental defects after implantation, although the differences were not correlated with the phenotype for peri-implantation development. Recent single-cell transcriptomic studies have highlighted the presence of heterogeneities in gene expression among early blastomeres^27,51–53^. The differences in transcriptomes between the two groups in our study might thus be explained in part by the successful development of blastomeres with a particular transcriptome.

The precise delineation of totipotent cells will require characterization of the concordance for developmental ability of single sister blastomeres isolated from the same parental embryos. Such characterization has not been addressed extensively with the exception of embryos at the two-cell stage in mice. Both blastomeres of embryos at the two-cell stage were thus shown to develop into monozygotic twin pups^54–56^, suggesting that each of the two blastomeres originating from the first cell division is totipotent. However, most monozygotic twin blastocysts have been found to differ with regard to the relative amounts of the ICM and TE^40^. A recent study further showed that both sister blastomeres are totipotent in only ~ 30% of embryos at the two-cell stage^57^. We have now found that 50% and 24% of embryos at the two- or four-cell stages, respectively, show concordance for the ability of isolated blastomeres to form a cavity, whereas concordance for this ability was virtually absent for embryos at the eight-cell or morula stages. Furthermore, we found that 13% of embryos at the two-cell stage show concordance for the ability to form EPI and PrE in two distinct compartments of the ICM, whereas concordance for this ability was essentially absent for embryos at the four-cell stage. These results indicate that the discordance in developmental ability of isolated single sister blastomeres of the same parental embryo starts to appear after the first cell division and becomes more pronounced after the second cell division. This discordance might be explained in part by interblastomere molecular differences. Recent studies have thus indicated that molecular differences among sister blastomeres already exist in mouse embryos at the four-cell stage. Arginine-26–dimethylated histone H3 (H3R26me2) was found to be distributed heterogeneously among 4CBs derived from the same parental embryo, with this difference being attributable to heterogeneous activity of CARM1 (coactivator-associated arginine methyl transferase 1)^58^. CARM1 was also found to increase the fraction of DNA bound to OCT4 and SOX2 as well as the expression of their downstream target genes, such as *Sox21*, in four-cell-stage embryos^59–61^. In addition, *Prdm14* was shown to be heterogeneously expressed among 4CBs derived from the same parental embryo, with interaction of PRDM14 with CARM1 promoting the formation of H3R26me2^62^. A PRDM14/CARM1–OCT4/SOX2–SOX21 signaling axis in embryos at the four-cell stage might thus bias progeny of blastomeres toward the ICM fate. Moreover, upstream regulators of this signaling axis, including the long noncoding RNA (lncRNA) LincGET and paraspeckles (nuclear structures formed by specific lncRNA-protein complexes), were found to be heterogeneously distributed between 2CBs derived from the same parental embryo and to act as the earliest known lineage regulators that bias cell fate in two-cell embryos^63,64^. These interblastomere molecular differences may result from unequal distribution of cell components when cells divide and may give rise to a deficiency of components required for development to term, leading to discontinuity of totipotency during progression from the zygote to two-cell and four-cell embryos. Given that embryos at the two- and four-cell stages are composites of totipotent and nontotipotent cells, further study of interblastomere molecular differences and of their relation to the developmental potential of isolated single blastomeres in such embryos is thus required to determine the molecular signature and basis of totipotency in mammals.

## Methods

### Mice

All animal experiments were approved by the animal ethics committee of Kyushu University and were performed in accordance with the ARRIVE guidelines and the regulations for animal experiments at Kyushu University. All mice were obtained from Charles River Laboratories Japan and were housed in individually ventilated cages under the specific pathogen–free condition. They were maintained on a 12-h-light, 12-h-dark cycle and at a room temperature of 23 to 25 °C and humidity of 40% to 60%.

### Isolation of spermatozoa and oocytes as well as in vitro fertilization

Metaphase II (MII) stage oocytes were collected from the oviducts of 8- to 9-week-old B6D2F1 female mice that had been treated with 7.5 IU pregnant mare serum gonadotropin and 7.5 IU human chorionic gonadotropin (ASKA Pharmaceutical), and spermatozoa were collected from the cauda epididymis of 15- to 20-week-old ICR male mice. For in vitro fertilization, MII stage oocytes were placed in human tubal fluid (HTF) medium (Irvine Scientific) supplemented with bovine serum albumin (BSA, Sigma-Aldrich) at 10 mg/ml and were exposed to spermatozoa in which capacitation had been induced by prior incubation for 1 h under a humidified atmosphere of 5% CO_2_, 5% O_2_, and 90% N_2_ at 37 °C in HTF medium supplemented with BSA at 4 mg/ml. At 5 hpi, fertilized oocytes with two pronuclei were washed and then cultured under a humidified atmosphere of 5% CO_2_, 5% O_2_, and 90% N_2_ at 37 °C in HTF medium containing BSA (4 mg/ml) and covered with liquid paraffin (Wako).

### Isolation and culture of blastomeres

Embryos at the one-cell (5 hpi), two-cell (24 hpi), four-cell (48 hpi), eight-cell (48 hpi), and morula (72 hpi, compacted 16-cell) stages were incubated for 100 s with 1% Actinase E (Kaken Pharmaceutical) in phosphate-buffered saline (PBS) under a humidified atmosphere of 5% CO_2_, 5% O_2_, and 90% N_2_ at 37 °C. After removal of the ZP, two- to eight-cell embryos were washed with HTF medium supplemented with BSA (4 mg/ml) before isolation of single blastomeres with the use of a narrow-bore glass pipette. Sixteen-cell embryos were incubated for 30 s with HTF medium supplemented with BSA (4 mg/ml) and EDTA (5 mM) under a humidified atmosphere of 5% CO_2_, 5% O_2_, and 90% N_2_ at 37 °C and were then washed with HTF medium supplemented with BSA (4 mg/ml) before isolation of single blastomeres with the use of a narrow-bore glass pipette. Isolated blastomeres were placed individually in the wells of a LinKID micro 25 plate (Dai Nippon Printing) and were cultured under a humidified atmosphere of 5% CO_2_, 5% O_2_, and 90% N_2_ at 37 °C in HTF medium containing BSA (4 mg/ml) and covered with liquid paraffin.

### Embryo transfer and C-section

Pseudoblastocysts (96 hpi) derived from single blastomeres were transferred surgically to the uterine horns of pseudopregnant or pregnant ICR female mice (8 to 12 weeks of age) at 2.5 dpc that had received the copulation plug from vasectomized or intact ICR male mice, respectively. C-section of pseudopregnant recipients was performed at E18.5.

### Evaluation of postnatal growth and fecundity test

The number of pups derived from embryos that had been transferred to pregnant recipients was counted at their delivery, and dams were allowed to nurse them together with their own progeny (ICR mice) for 21 days after delivery. The number of pups to be nursed was adjusted to six per dam, and weaned pups were counted at 21 days after birth. Pups derived from transferred embryos (black eyes and a dark coat) were distinguished from ICR pups (red eyes and a white coat) by eye and coat color. Eight-week-old female and male mice derived from isolated blastomeres were housed with 8-week-old B6D2F1 counterparts and evaluated for their fecundity over a 2-month period.

### Immunostaining of embryos and ESCs

Immunofluorescence staining was performed as described previously^65^. In brief, pseudoblastocysts (96 or 120 hpi) or ESCs were fixed for 20 min at room temperature with 4% paraformaldehyde in PBS, washed with PBS containing 0.05% Tween 20 (PBST), and permeabilized for 30 min at room temperature with 0.4% Triton X-100 in PBS. They were then washed with PBST before incubation first for 1 h at room temperature with 1% BSA in PBST and then overnight at 4 °C with rabbit anti-CDX2 (1:1000 dilution, Abcam ab76541), mouse anti-OCT4 (1:500 for embryos and 1:250 for ESCs, Santa Cruz sc-5279), rabbit anti-NANOG (1:200, Abcam ab80892), mouse anti-GATA4 (1:200, Santa Cruz sc-25310; 1:1000, Santa Cruz sc-25310AF548), or mouse anti-DAB2 (1:1000, Santa Cruz sc-136964AF488) in the same solution. They were again washed with PBST, incubated for 1 h at room temperature with appropriate Alexa Fluor 488– or Alexa Fluor 568–conjugated secondary antibodies (Molecular Probes–Invitrogen), washed with PBST, and mounted in SlowFade Gold antifade reagent (Molecular Probes–Invitrogen) containing Hoechst 33342 (5 μg/ml) or RedDot2 (1:200, Biotium). Fluorescence images were acquired at multiple 1-μm intervals in the *z*-axis with the use of a confocal microscope (CV1000, Yokogawa).

### Blastocyst outgrowth assay

Pseudoblastocysts (96 hpi) derived from single blastomeres were transferred individually to the wells of 24-well plates that had been coated with 0.1% gelatin and were cultured for 5 days under a humidified atmosphere of 5% CO_2_ at 37 °C in Dulbecco’s modified Eagle’s medium (DMEM) containing HEPES (Wako 048–30,275) and supplemented with 15% fetal bovine serum (Hyclone SH30396.03), penicillin–streptomycin at 100 U/ml (Wako 168-23191), and 10 µM 2-mercaptoethanol (Wako 131-14572).

### Derivation of ESCs from single blastomeres

Pseudoblastocysts (96 hpi) derived from single blastomeres were transferred individually to the wells of 24-well plates that had been coated with 0.1% gelatin and were cultured for 2 days under a humidified atmosphere of 5% CO_2_ at 37 °C in ESC culture medium consisting of Glasgow modified essential medium (GMEM, Wako 078-05525) supplemented with 10% fetal bovine serum, Glutamax (Thermo Fisher Scientific 35050-061), 1 mM sodium pyruvate (Wako 531-16551), penicillin–streptomycin at 100 U/ml, nonessential amino acids (Wako 139-15651), 0.1 mM 2-mercaptoethanol, LIF at 1000 U/ml (Wako 195-16053), and inhibitors of the kinases MEK (PD0325901 at 0.5 μM, Wako 162-25291) and GSK3 (CHIR99021 at 1.5 μM, Wako 034-23103). Pseudoblastocysts attached to the plates were then allowed to undergo outgrowth for 14 days under a humidified atmosphere of 5% CO_2_ at 37 °C in the same culture medium. The outgrowths were isolated by exposure to 0.25% trypsin–EDTA (Wako 209-16941) and subjected to disaggregation by pipetting before transfer to 12-well plates coated with 0.1% gelatin. The ESCs were cultured and passaged six times before assessment of their pluripotency by immunostaining with anti-OCT4 and anti-NANOG.

### Transcriptomic analysis of pseudoblastocysts derived from single blastomeres

Libraries were constructed according to the CEL-Seq2 protocol^66^ and were sequenced with an Illumina HiSeq 1500 instrument. The reads were aligned to the reference genome GRCm38 with HISAT2 software (ver. 2.2.6)^67^. Unique molecular identifier (UMI) counts per gene were generated by HTSeq (ver. 0.6.1)^68^, with a consensus read being counted for each group of duplicate reads sharing the same UMI tag. The UMI counts were normalized as described previously^69^ and were entered into DESeq2 (ver. 1.10.1)^70^ to determine differentially expressed genes (at an FDR of < 0.1). The data generated in this study are available in the Gene Expression Omnibus (GEO) database of NCBI under the accession number GSE163427.

### Statistical analysis

Data are presented and were analyzed as described in figure legends. Simultaneous 95% CIs for differences in proportions were determined as described^71^. A *P* value of < 0.05 was considered statistically significant.

## Supplementary Information

Supplementary Information.
