# Supplementary material for: Totipotency of mouse zygotes extends to single blastomeres of embryos at the four-cell stage
Source: Sci Rep. 2021 May 27;11:11167. doi: 10.1038/s41598-021-90653-1 (PMC8160171; doi:10.1038/s41598-021-90653-1)

Supplementary Information

Totipotency of mouse zygotes extends to single blastomeres of embryos at the four-cell stage

Marino Maemura, Hiroaki Taketsuru, Yuki Nakajima, Ruiqi Shao, Ayaka Kakihara, Jumpei Nogami, Yasuyuki Ohkawa & Yu-ichi Tsukada*

*Corresponding author: ytsukada@ifrc.kyushu-u.ac.jp

Supplementary Tables S1−S5
Supplementary Figures S1−S3

Supplementary Table S1. Development of embryos derived from single blastomeres in the uterus of pseudopregnant female mice.

Female number	Blastomere stage	No. of pseudoblastocysts transferred	No. of viable pups	No. of absorbed embryos
1	1CZ	20	7	3
2	1CZ	20	0	0
3	1CZ	20	5	8
4	1CZ	20	4	7
5	1CZ	20	3	2
6	1C	20	2	6
7	1C	20	6	3
8	1C	20	2	2
9	1C	20	0	0
10	1C	20	4	2
11	2CB	20	2	0
12	2CB	20	4	8
13	2CB	20	2	0
14	2CB	20	1	0
15	2CB	20	2	1
16	4CB	20	0	0
17	4CB	20	0	0
18	4CB	20	0	0
19	4CB	20	0	0
20	4CB	20	0	0
21	8CB	20	0	0
22	8CB	20	0	0
23	8CB	20	0	0

24	8CB	20	0	0
25	8CB	20	0	0

Data correspond to those in Figure 3a and Supplementary Figure S1a–c. Embryo transfers were performed on at least three different days for each blastomere stage. Absorption was evident as implantation sites without embryos.

Supplementary Table S2. Development of embryos derived from single blastomeres at the four- or eight-cell stage in the uterus of pseudopregnant female mice.

Female number	Blastomere stage	No. of pseudoblastocysts transferred	No. of viable pups	No. of absorbed embryos
1	4CB	30	1	6
2	4CB	30	0	0
3	4CB	30	0	0
4	4CB	30	0	0
5	4CB	30	1	1
6	8CB	30	0	0
7	8CB	30	0	0
8	8CB	30	0	0
9	8CB	30	0	0
10	8CB	30	0	0

Data correspond to those in Figure 3b and Supplementary Figure S1d–f. Embryo transfers were performed on at least three different days for each blastomere stage. Absorption was evident as implantation sites without embryos.

Supplementary Table S3. Development of embryos derived from single blastomeres in the uterus of pregnant female mice.

Female number	Blastomere stage	No. of pseudoblastocysts transferred	No. of viable pups derived from single blastomeres	No. of viable pups derived from foster female
1	1CZ	20	3	5
2	1CZ	20	10	1
3	1CZ	20	10	5
4	1CZ	20	6	5
5	1CZ	20	10	4
6	1C	20	1	7
7	1C	20	1	8
8	1C	20	0	7
9	1C	20	3	5
10	1C	20	4	8
11	2CB	20	2	7
12	2CB	20	2	10
13	2CB	20	3	11
14	2CB	20	4	8
15	2CB	20	0	8
16	4CB	20	0	8
17	4CB	20	0	1
18	4CB	20	0	4
19	4CB	20	0	4
20	4CB	20	0	3

Data correspond to those in Figure 3d and Supplementary Figure S2a. Embryo transfers were performed on at least three different days for each blastomere stage.

Supplementary Table S4. Development of embryos derived from single blastomeres at the four- or eight-cell stage in the uterus of pregnant female mice.

Female number	Blastomere stage	No. of pseudoblastocysts transferred	No. of viable pups derived from single blastomeres	No. of viable pups derived from foster female
1	4CB	30	1	12
2	4CB	30	2	5
3	4CB	30	1	10
4	4CB	30	1	1
5	4CB	30	0	3
6	8CB	30	0	12
7	8CB	30	0	14
8	8CB	30	0	11
9	8CB	30	0	8
10	8CB	30	0	9

Data correspond to those in Figure 3e and Supplementary Figure S2b. Embryo transfers were performed on at least three different days for each blastomere stage.

Supplementary Table S5. Fertility of mice derived from single blastomeres.

Blastomere stage	No. of females tested	No. of females that produced offspring	No. of males tested	No. of males that produced offspring
1CZ	4	4	3	3
1C	3	3	3	2
2CB	3	3	3	3
4CB	4	4	1	1

Supplementary Figures

Supplementary Figure S1. 4CB embryos possess the potential to develop to term. (**a**, **b**) The number of viable pups (**a**) or absorbed embryos (**b**) for each foster female in Figure 3a. (**c**) The rate of absorbed embryos determined as the proportion of implantation sites without viable embryos among all transferred pseudoblastocysts in Figure 3a is shown in the left panel, with the difference in the rates of absorbed embryos among the different types of embryo being shown together with its simultaneous 95% CI in the right panel. The *P* values were determined with the two-sided Fisher's exact test followed by adjustment with the BH method (FDR = 0.05). $^*P < 0.01$, $^{**}P < 0.001$. *n* indicates the number of pseudoblastocysts transferred. (**d**, **e**) The number of viable pups (**d**) or absorbed embryos (**e**) for each foster female in Figure 3b. (**f**) The rate of absorbed embryos in Figure 3b is shown in the left panel, with the difference in the rates of absorbed embryos between 4CB and 8CB embryos being shown together with its 95% CI in the right panel. The *P* value was determined with the two-sided Fisher's exact test. *n* indicates the number of pseudoblastocysts transferred. (**g**, **h**) The weight of viable pups at E18.5 (**g**) and of their placentas (**h**) for Figure 3a and 3b are shown as box-and-whisker plots (Tukey style). The *P* values were determined with the Kruskal-Wallis test (**g**) or with the Kruskal-Wallis test followed by the Steel-Dwass test (**h**). *n* indicates the number of viable pups (**g**) or their placentas (**h**).

Supplementary Figure S2. 4CB embryos give rise to fertile adults. (**a**, **b**) The number of viable pups for each foster female in Figure 3d and 3e, respectively. (**c**, **d**) Body length (**c**) and weight (**d**) of 3-week-old weaned female (left panels) and male (right panels) pups from Figure 3d and 3e are shown as box-and-whisker plots (Tukey style). The *P* values were determined with the Kruskal-Wallis test (left panels) or with the Kruskal-Wallis test followed by the Steel-Dwass test (right panels). $^*P < 0.01$. *n* indicates the number of weaned female mice (left panels) or male mice (right panels). (**e**) Representative image of a female mouse derived from a 4CB embryo and her pups.

Supplementary Figure S3. Proportion of two-cell or four-cell parental embryos that show concordance among sister blastomeres for the ability to form presumptive functional EPI. 2CB and 4CB pseudoblastocysts cultured *in vitro* up to 120 hpi as in

Figure 7b were subjected to immunofluorescence staining with anti-NANOG and anti-GATA4 for evaluation of EPI and PrE formation. The proportion of pseudoblastocysts that showed signals for NANOG in the ICM out of all pseudoblastocysts derived from each twin or quadruplet set of blastomeres is represented by x. Parental embryos were classified into three categories on the basis of this proportion. *$P < 0.01$, two-sided Fisher's exact test. n indicates the number of twin or quadruplet blastomere sets (parental embryos).

Supplementary Figure S1

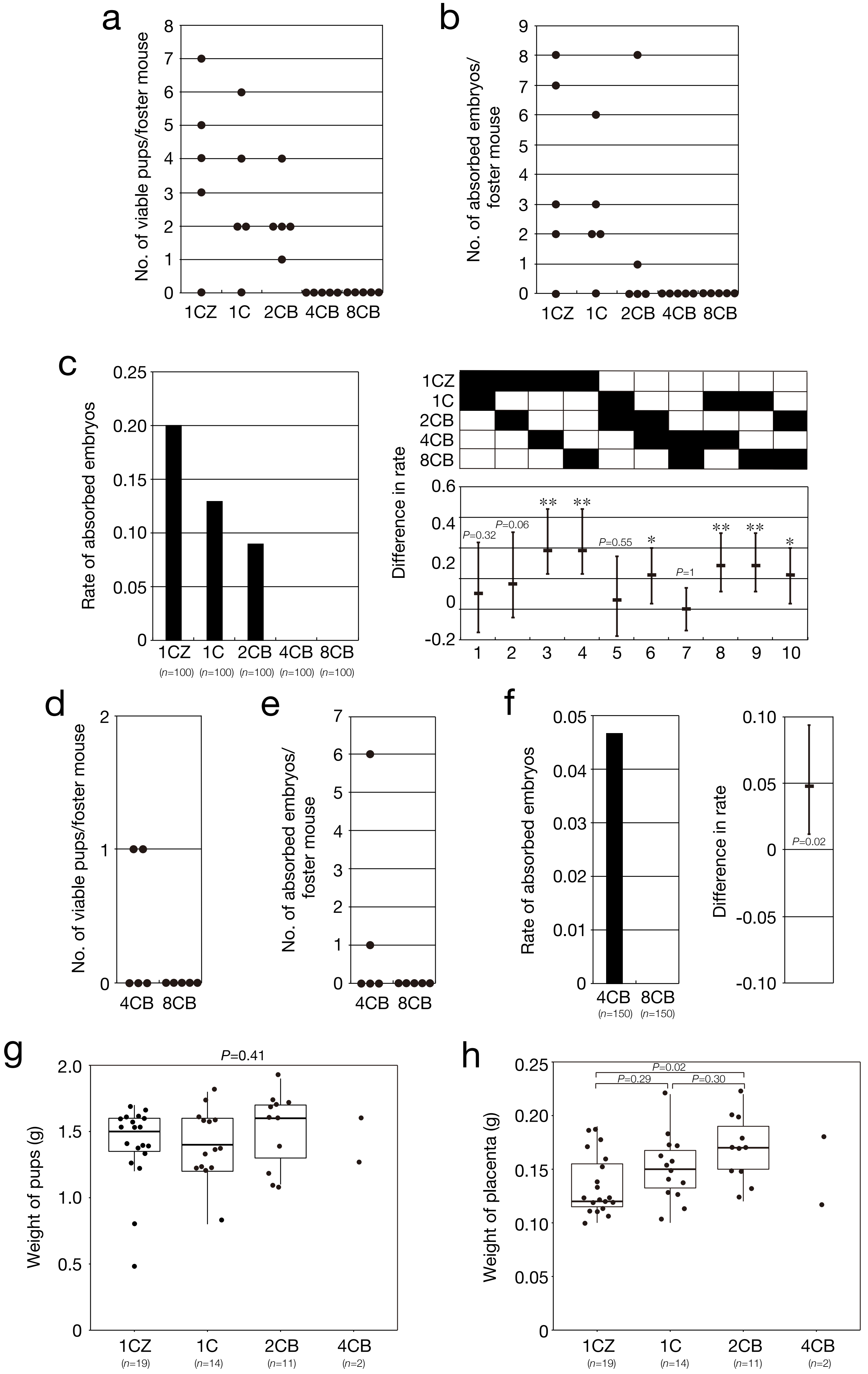

Supplementary Figure S2

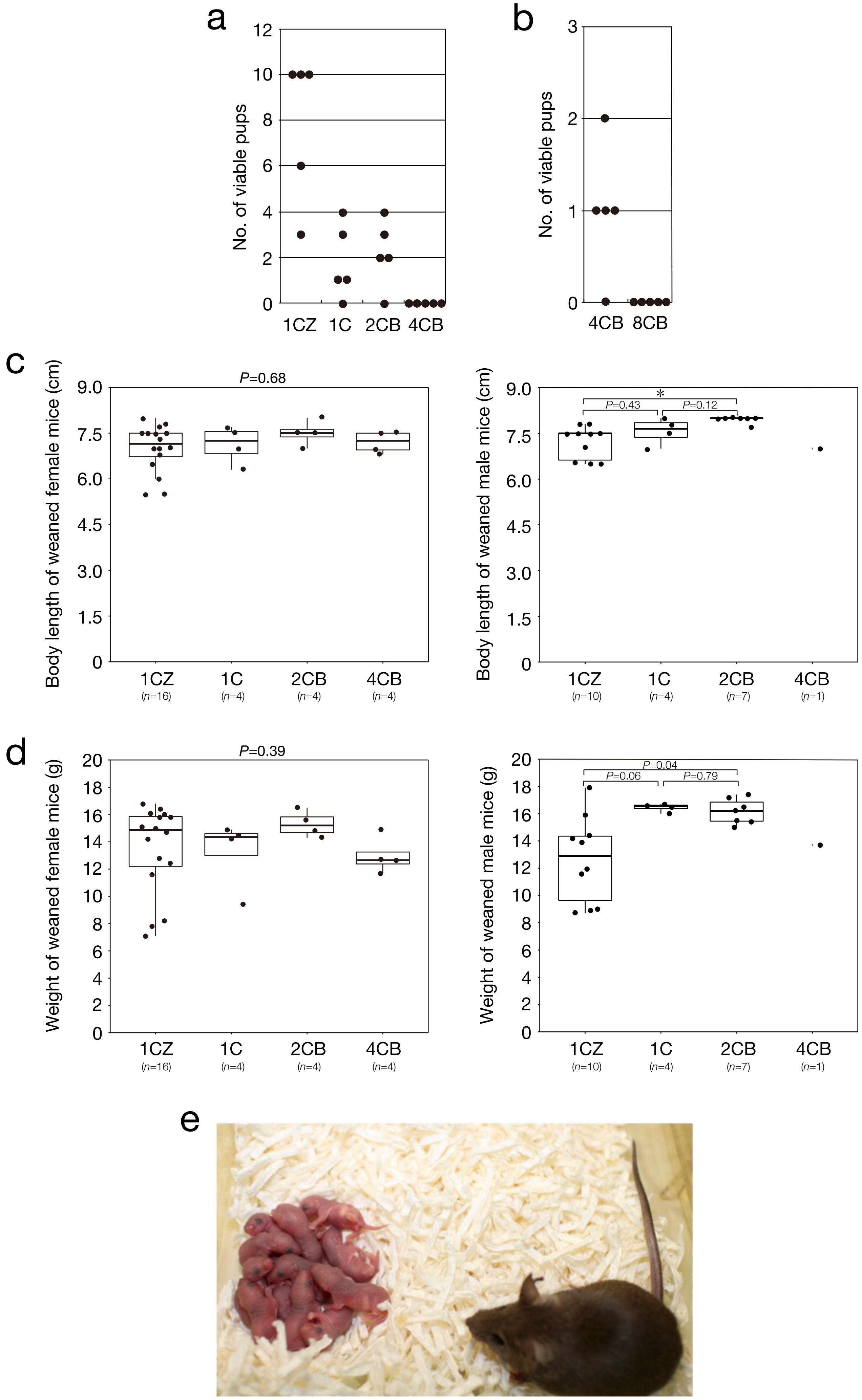

Supplementary Figure S3

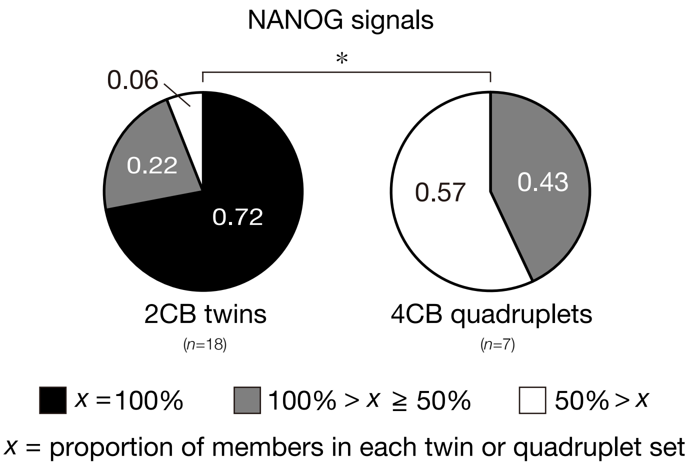

Supplement: Supplementary file 1 — Supplementary Information. [file 41598_2021_90653_MOESM1_ESM.pdf]
